# The Cognitive Dimension and the Affective Dimension in the Patient’s Experience

**DOI:** 10.3389/fpsyg.2019.02177

**Published:** 2019-09-25

**Authors:** Pedro Reinares-Lara, Alfredo Rodríguez-Fuertes, Blanca Garcia-Henche

**Affiliations:** ^1^Department of Business Economics, Universidad Rey Juan Carlos, Madrid, Spain; ^2^Department of Economy and Business Management, Universidad de Alcalá, Madrid, Spain

**Keywords:** patient experience, patient journey, facial expression analysis, emotions, satisfaction, consumer neuroscience, customer experience

## Abstract

This article deals with the experience of the specific client of health services, that is, the patient. Satisfaction questionnaires are usually applied to assess patient experience. However, this tool provides only a cognitive evaluation; it does not afford an affective dimension of the experience. The objective of the present study is to verify the relationship between the cognitive dimension of patient experience, collected through questionnaires, and the affective dimension, derived from the analysis of neurophysiological data. We propose a novel methodology that integrates physiological data collected by facial expression analysis to identify patients’ emotions. A first, qualitative procedure was carried out to define the patient journey. This was recorded on video and later used in the experiment. The experiment collected information from the participants using two techniques. First, as they viewed the videos, facial expression analysis (FEA) was applied to assess their responses. Second, after they watched the videos, traditional questionnaires were presented. The results provided by the two techniques were then compared. The results show that there is no relationship between the emotional valence reported by questionnaires and the neurophysiological data. This reflects the two different dimensions of the experience, one cognitive and the other affective. Both facilitate the understanding of patient satisfaction.

## Introduction

In recent years, there has been a growing interest in the study of the customer experience, especially in terms of emotions. There has been much analysis of consumer satisfaction with services and the variables that affect it. Although satisfaction is a generally well-understood concept, there is no consensus on its nature, nor on its evaluation (Giese and Cote, 2000; Villodre et al., 2014).

While it is necessary for any organization to have satisfied customers, it is a priority objective in the health sector. Healthcare is a critical context due to its unpredictable situations, demanding clients, workload, and intrinsic organizational complexity. The need for healthcare quality improvement in a period of increasing financial and service pressures requires for financial performance and productivity not to negatively impact on service quality (Bruno et al., 2017). Private entities have shown that satisfied customers are more likely to be loyal to the supplier of the service that gave the satisfaction (Bendall-Lyon and Powers, 2004; Sofaer and Firminger, 2005; Rundle-Thiele and Russell-Bennett, 2010), and to recommend that service provider (Bendall-Lyon and Powers, 2004). Satisfaction also influences clinical outcomes. Studies have shown that patients who reported themselves as being more satisfied with their care are more positive, compliant, cooperative, and more likely to increase their participation in prescribed medical and pharmaceutical treatments (Dubé and Menon, 1998; Sofaer and Firminger, 2005).

According to some authors, service quality is mainly cognitive, whereas satisfaction is a more complex concept that includes both cognitive and affective components (Oliver, 1993). It is possible that both aspects might be defined by examining more closely recognized quality dimensions, cognitive satisfaction and affective satisfaction (Cronin and Taylor, 1992; Vinagre and Neves, 2008).

## Conceptual Framework

### Patient Experience

There has been a rapid acceptance of the use of the term “patient experience”: as evidence, it is now a top priority for healthcare management (Wolf et al., 2014). In a sector, such as healthcare, in which the internal perspective has predominated in terms of designing work processes and assessing service quality, knowing how the patient experiences the process is an opportunity to improve this experience.

The Beryl Institute (2015) defined patient experience as “the sum of all interactions, shaped by an organization’s culture, that influence patient perceptions across the continuum of care.” This definition reflects the multidimensional nature of patient experience previously included in the healthcare literature (Gentile et al., 2007; The King’s Fund, 2010), comprising sensory, cognitive, and emotional components (Fulbright et al., 2001).

Patient satisfaction is a commonly used indicator for measuring patient experience in healthcare. However, there are methodological weaknesses regarding the techniques used to address the issue of experience (Sofaer and Firminger, 2005), as the individual is considered to be eminently rational and capable of processing all the data from the experience to express his or her opinion at a given time. For this reason, it is believed that there is currently no reliable method for collecting data on patient experience (Coulter et al., 2014).

### Double Dimension of Satisfaction

The complexity of the process leading to satisfaction with health services involves diverse phenomena associated with both the cognitive and emotional domains (Vinagre and Neves, 2008). Several authors have confirmed that satisfaction has a double dimension:

(1) First, there is a cognitive evaluation side, which is a result of a cognitive process in which the patient considers the positive and negative aspects of different components of a service, either evaluating the perceived result alone, or comparing it against a standard (Liljander and Strandvik, 1997). From this perspective, satisfaction with a healthcare experience is the result of the accumulation of independent evaluations of different factors, such as medical staff, environment, service, etc. (Westbrook and Oliver, 1991).

(2) A second, affective side, considers subjective elements by capturing feelings or emotions generated in the relationship between the patient and the healthcare provider (Cronin and Taylor, 1992; Oliver, 1993; Liljander and Strandvik, 1997; Gill and White, 2009) that are beyond the patient’s conscious control.

Healthcare organizations today focus on cognitive assessments and neglect the emotions experienced by the patient. However, it has been demonstrated that emotional aspects not only have an impact on patient satisfaction, they also influence clinical outcomes (Dubé and Menon, 1998; Sofaer and Firminger, 2005).

For these reasons, in recent years some researchers have advocated the need to address satisfaction from a dual cognitive-affective approach (Westbrook and Oliver, 1991; Wirtz and Bateson, 1999; Villodre et al., 2014), considering cognitive and affective responses as distinct, since each has a separate influence on satisfaction formation (Oliver, 1993; Liljander and Strandvik, 1997). It is also likely that this approach will help to define the concepts of service quality and satisfaction (Cronin and Taylor, 1992; Vinagre and Neves, 2008).

It is possible that the measurement of the affective-subjective component of the patient satisfaction construct still lacks precision (Crow et al., 2002), which would render the studies undertaken into the subject purely exploratory (Gill and White, 2009).

### Satisfaction Measurement

The literature review shows that the concept of emotion has attracted the interest of researchers in understanding the background and results of customer satisfaction. Hunt (1977) considered satisfaction as the evaluation of an emotion. Emotions are complex phenomena that arise when judging an event, in which various factors (physiological, behavioral, expressive, and subjective feelings) intervene, and that influence the individual’s decision-making and judgment.

There are two main theoretical approaches to measure emotional subjective experience: the categorical approach, which studies basic emotions, and the dimensional, which looks at emotional dimensions and the degree to which these dimensions are perceived. Researchers who take the categorical approach seek to determine which of a given set of basic emotions (e.g., fear, anger, joy, sadness, acceptance, disgust, expectancy, and surprise) a subject is feeling (Pelegrín-Borondo et al., 2005). Some researchers who take the dimensional approach believe that emotional state can be determined exclusively based on levels of arousal and pleasure. The present study takes this second line. This method of obtaining self-reports of emotional feelings is simple and straightforward and generally quite reliable (Scherer, 2005).

**Figure 1:**
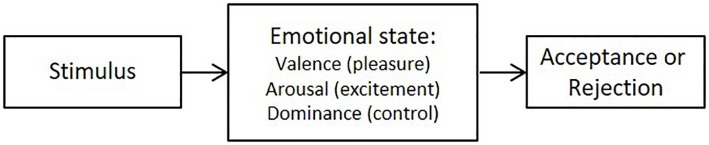
PAD model. Source: Mehrabian and Russell (1974).

To examine the affective component, we used the PAD (pleasure-arousal-dominance) model developed by Mehrabian and Russell (1974). This explains that an individual experiences his or her emotions through the combination of three continuous psychophysiological bipolar variables: valence (or pleasure), activation (or excitement), and dominance (or control) (Figure 1).

1. Valence (pleasure)

Valence refers to the subjective, emotional value evoked by the stimulus (Russell, 1980). This value classifies participants’ emotions as positive (when they provoke a pleasant state), or negative (when they provoke displeasure). The former have associated approach behaviors, while the latter have been described as motivations to avoid and generate rejection or withdrawal behaviors (Cacioppo and Gardner, 1999). That is, in the case of negative valence, the behavior of the individual will not be repeated.

2. Arousal

Arousal, activation or excitation, refer to the affective experience of energy levels. When an individual experiences a high level of energy, this evokes high arousal, whereas the experience of low levels of energy is associated with low arousal. Depending on how stimulating the environment is, the individual’s behavior is aimed at consciously increasing or reducing activation levels. When activation levels are low, any increase in environmental stimuli will be pleasurable, and any reduction will be unpleasant. Moderate activation levels result in a pleasant experience, while boring or stressful stimuli produce aversive experiences (Reeve, 2015).

3. Dominance

The dominance dimension refers to the degree of control exerted by the stimulus on the individual. It offers lower discrimination than valence and arousal and is not always used in research (Posner and Rothbart, 1998; Setz et al., 2009).

### Evaluating Patient Experience

First, it is necessary to recognize the lack of consensus not only on a definition of satisfaction, but also on its evaluation (Giese and Cote, 2000; Villodre et al., 2014).

To collect data about patients’ experiences, quantitative techniques are generally used, as are, less commonly, qualitative techniques. Quantitative techniques are based on questionnaires used to investigate patients’ perceptions (Granado et al., 2007; Val-Jiménez et al., 2017). These have emerged as valid instruments for measuring the cognitive component of satisfaction, but are used less so for measuring experience and emotions or the “emotional experience” referred to by Frijda and Mesquita (1998), Granado et al., (2007), and Val-Jiménez et al., (2017).

Qualitative techniques offer a greater potential to uncover more in-depth facts about healthcare services (Ofili, 2014) and help researchers gather more customer data. For this reason, the use of qualitative research techniques is recommended to identify and understand the principal determinants of satisfaction with healthcare services (Liljander and Strandvik, 1997; Losada and Rodríguez, 2007), as quantitative research alone does not seem capable of representing patients’ experiences (Lees, 2011). Some researchers argue that using combined quantitative and qualitative methodologies offers a better solution than other techniques (Losada and Rodríguez, 2007; Lees, 2011; Ofili, 2014).

There has been a progressive incorporation of neuroscience techniques into marketing research over the last decade (Ausin et al., 2017). These techniques, for example, FEA, one of the most valued methods, help to study emotions. There is no research into patient experience that applies neurophysiological data-gathering techniques of responses outside the patient’s conscious control. This offers the opportunity to incorporate new methodologies capable of providing data on the emotional side of patient experience.

Against this background, the present study aims to verify the possible relationship between data reported through questionnaires, which provide a cognitive, conscious assessment, and neurophysiological data, which reflect affective processes outside the patient’s conscious control.

## Materials and Methods

In order to achieve the research objective, a two-phase process was adopted: a first exploratory phase and an experimental phase. The study was approved by the internal review board of the laboratory of neuromarketing at the Universidad Rey Juan Carlos. The participants signed a written informed consent in accordance with the Declaration of Helsinki.

### Exploratory Stage

In the exploratory stage, qualitative methods were used, with 3 focus groups and 14 in-depth interviews, collecting data from patients, health professionals, and health quality experts.

The objectives of this first stage were to delve into the emotional aspects of the care experience and to describe the patient journey (PJ). Specifically, we wanted to reproduce the PJ of individuals undergoing inguinal hernia surgery, the process subsequently used in the experiment. We chose this procedure because it is one of the most common in the Spanish health system and it involves different levels of care: (1) primary care (PC), (2) specialized care (SC), and (3) hospitalization and discharge (H).

### Experiment

The experiment consisted of studying the emotional value of a sample of individuals during the simulation of a surgical intervention process. We showed the participants a video of patients going through the process of an inguinal hernia repair surgery in a health facility, including their interactions with the healthcare professionals.

The PJ was divided into three stages on a 3-min video, in order to collect data at the end of each stage and analyze them independently. These stages correspond to each of the care process levels, PC, SC, and H. The videos were shown to the participants to collect data on their emotions at specific moments, on the basis that the patients’ would experience emotions as if the procedure was being applied to them at that moment. This design allows customer perceptions and feelings to be examined throughout a simulated event (Suomala et al., 2012).

For the emotional assessment of the experience, a double system was used: (1) a SAM questionnaire was presented to the participants after they had watched the videos; this provided data of their emotional valence and arousal and (2) their neurophysiological variables were monitored by a facial expression analysis team as they watched the videos.

Given that there is a link between the valence dimension of emotions and consumer satisfaction (Phillips and Baumgartner, 2002), the participants were asked, after watching the three videos, to assess their satisfaction with the process in a questionnaire, in order to corroborate the possible relationship.

The study design, using two types of techniques, helped meet the methodological aim of comparing the physiological measures obtained by facial expression analysis with the results from the SAM questionnaire.

In the service sector, studies frequently use self-completion questionnaires combined with data collected by neuroscience devices (Chih-Chien and Ming-Chang, 2014; Vance et al., 2014).

### Self-Assessment Manikin Questionnaire

The Self-Assessment Manikin (SAM) questionnaire was used to obtain data from individuals after they watched the videos, by which means, self-reported emotional experiences at each care level (PC, SC, and H) were collected.

The SAM questionnaire was developed by Bradley and Lang in 1994, based on Mehrabian’s three-dimensional PAD scale (Mehrabian, 1996). The SAM questionnaire is a widely accepted tool for assessing emotional responses (Greenwald et al., 1989). It is a self-administered questionnaire that uses a non-verbal assessment scale to directly measure the pleasure and arousal associated with an individual’s affective reaction to a stimulus.

**Figure 2:**
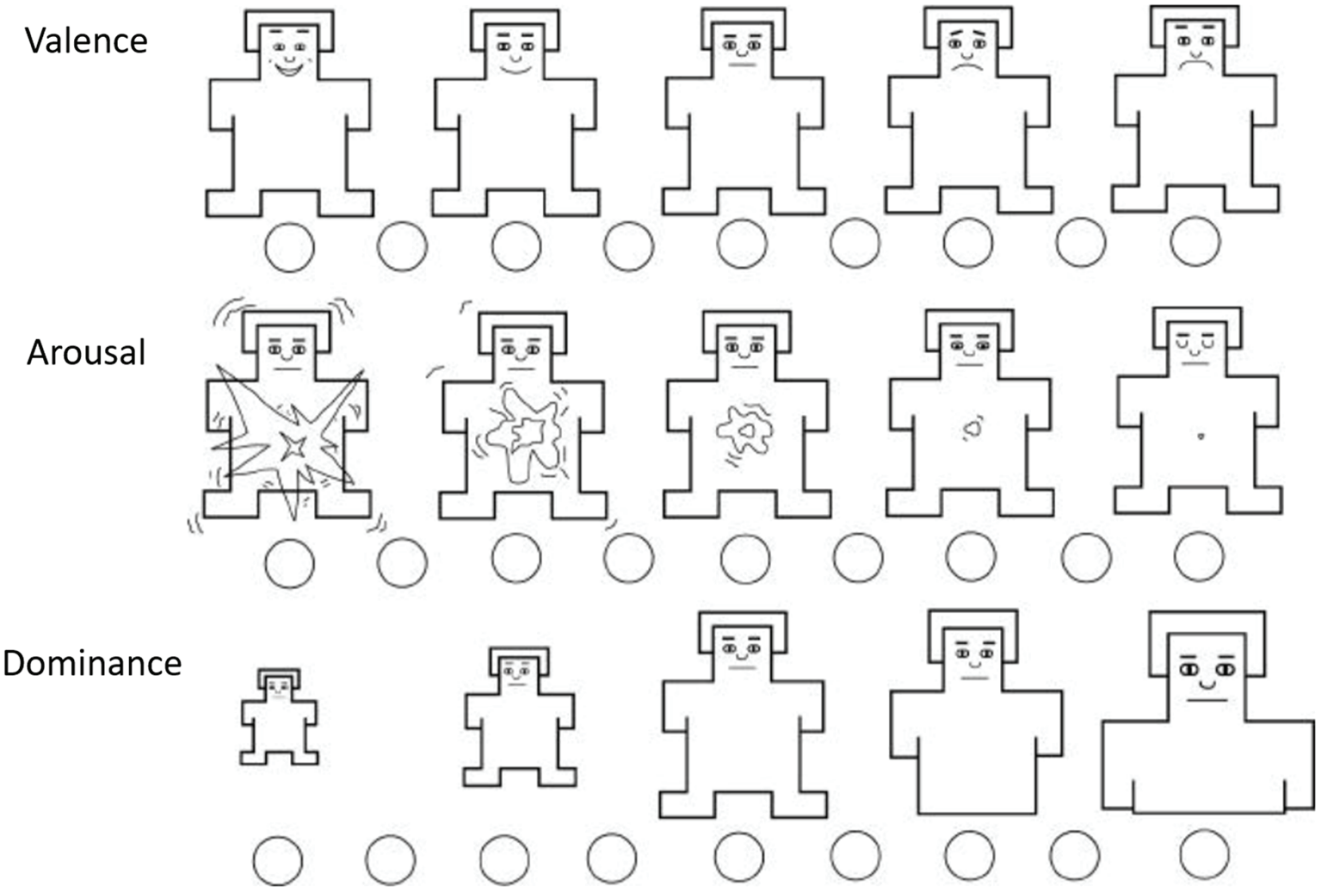
SAM questionnaire. Source: Bradley and Lang (1994).

The SAM measurement used consisted of a series of human figures, for each dimension, on a graduated valence-intensity scale (from a smiling to an unpleasant looking figure) and an arousal scale (from an excited to a relaxed figure) (Figure 2). Graphical evaluation techniques reduce the individual’s effort in verbalizing emotions. The dominance variable was not examined in the experiment because of its lower level of discrimination and its positive correlation with valence.

The SAM questionnaire has been used in health research (Jayanti and Whipple, 2008) and has also been used to rate the affective dimensions of valence and arousal of individuals while watching videos (Setz et al., 2009; Handayani et al., 2015).

### Facial Expression Analysis

As a complement to the SAM questionnaire, and in order to overcome the limitations of self-reported data, we used a neurophysiological measurement technique: facial expression analysis (FEA).

FEA captures emotions outside the patient’s conscious control that have their origins in the autonomous nervous system. The technique is based on the correlation found by Ekman and Friesen (1971) between emotions and facial muscle movements. FEA identifies the visible movements of facial muscles and is a novel technique, not so much because of its origin, as the first studies into facial coding systems were developed by Ekman and Friesen (1978), but because of the incorporation of new computer applications that allow the automatic coding of expressions.

For the present study, we used Affectiva-Affdex software integrated into the iMotions platform; this identified the emotions evoked and generated the valence indicator, which measured the positive and negative nature of the individual’s experience (iMotions, 2018).

**Figure 3:**
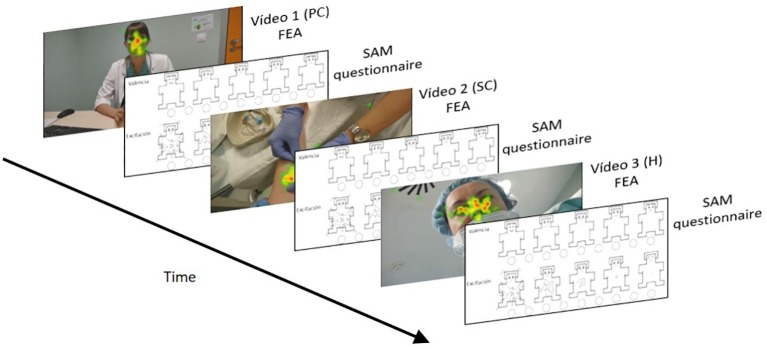
Experimental schema.

The experiment was conducted in accordance with the schema shown in Figure 3. Immediately after watching the video, the SAM questionnaire was administered. Thus, each participant assessed their level of satisfaction with the entire process, as if they had been the patient.

A total of 60 people participated in the experiment (mean age: 21.7 years; SD: 2.21; 50% men/50% women). This sample size is larger than that used in other FEA studies. The participants were between 18 and 65 years old, randomly recruited through a volunteer database encompassing all ages. The eligibility criteria were as follows: they had to be native Spanish language speakers, public health service users, not having undergone surgery in the previous 12 months, and who had never previously suffered from an inguinal hernia given that this was the reason for the PJ.

## Results

Data from the exploratory stage confirmed the high emotional burden that the experience of healthcare has for the patient and his or her family. The patients direct their demands towards those aspects that reduce anxiety (information) and fear (a trust relationship with the professional which may help them understand what to expect). The experience is more intense in more complex processes, in which referral to other levels is made during the hospital stay.

Although users generally expressed high levels of satisfaction with healthcare services, negative comments appeared when they referred in more detail to their experiences. Throughout the care process, negative emotions (anxiety at lack of information, fear of the results, sadness, etc.) were evident, but only in some cases did positive emotions appear (hope of a recovery of total or relative normality).

As a result, a detailed reconstruction of the patient journey of individuals undergoing inguinal hernia repair was achieved, based on 22 specific moments, from the appearance of the first symptoms of the problem, through the surgical intervention, and on to discharge (Table 1).

**Table 1:** Patient journey, inguinal hernia repair surgery.

Step	Moments
1. Primary care (PC)	1. First symptoms 2. The patient makes an appointment *via* the Internet to see a general practitioner at the health center 3. The patient goes to the health center 4. The patient enters the practitioner’s consultation room 5. After listening to the patient, she/he diagnoses an inguinal hernia and refers the patient to a specialist 6. The patient makes an appointment at the health center to see the specialist
2. Specialized care (SC)	A few weeks later: 7. The patient arrives at the specialism center for a consultation 8. S/he enters the specialist’s consultation room. The specialist confirms the inguinal hernia diagnosis and refers the patient for testing 9. Diagnostic tests 10. The patient goes with the results to the specialist who confirms the need for the intervention 11. Consultation with the preanesthetic physician
3. Hospitalization and discharge (H)	A few weeks later: 12. The patient arrives at the hospital admission desk 13. An assistant from the Admissions Department talks with the patient 14. S/he accompanies the patient to the room 15. The assistant shows the patient the room 16. A nurse provides information about the intervention 17. The patient goes to the operating theater 18. After entering the operating theater, the patient is anesthetized 19. The patient awakes from the anesthesia 20. The physician gives him/her the report and confirms the discharge 21. The patient leaves the hospital 22. A few days later, the patient goes for a check-up at the health center

This *PJ* was the basis for developing the video materials used in the experiment, which were recorded by professional teams in real environments with the participation of healthcare professionals.

### Self-Assessment Manikin Questionnaire Scores

**Figure 4:**
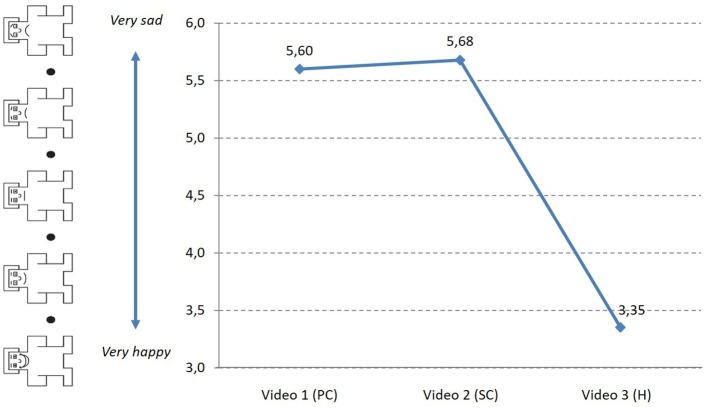
Evolution of valence (SAM) during the *PJ.*

The valence indicator showed the emotional values (positive or negative) evoked by the stimulus (Figure 4); these indicate that valence increases slightly from 5.60 to 5.68 between Stage 2 (SC) and Stage 1 (PC). It decreases considerably later, in Stage 3 (H), to 3.35, indicating an increase in patient happiness at the end of the process.

**Figure 5:**
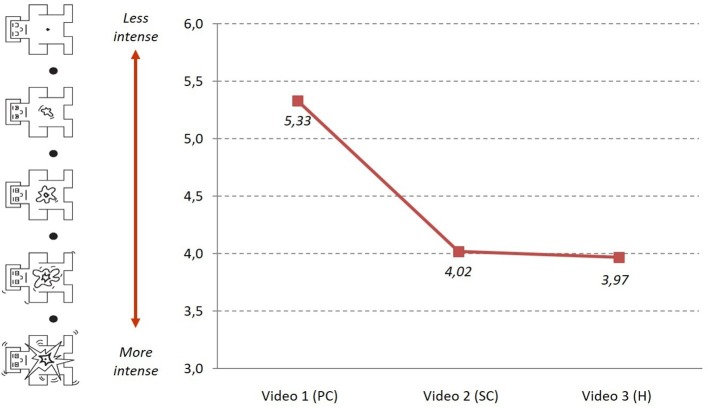
Evolution of arousal (SAM) during the *PJ.*

The arousal indicator, which reveals the degree of intensity with which these emotions are experienced (Figure 5), reports an increase, especially between Stages 1 (5.33) and 2 (4.02), and a decrease between Stages 2 and 3 (3.97).

The results showed a low inverse linear correlation between valence and arousal at Stage 1 (*r* = −0.268, *p* = 0.038) and at Stage 2 (*r* = −0.261, *p* = 0.044), but not at Stage 3 (Table 2). That is, arousal is less intense at higher valence levels (sadder) in Stages 1 and 2.

**Table 2:** Correlations of valence and arousal during each video (SAM).

Correlation between	Pearson correlation	Sig. (bilateral)
1. Valence and arousal Stage 1 (PC)	−0.268	**0.038**
2. Valence and arousal Stage 2 (SC)	−0.261	**0.044**
3. Valence and arousal Stage 3 (H)	−0.013	0.924

*Bold values represent the significant difference*.

### Comparison Between Self-Assessment Manikin Questionnaire and Satisfaction Level

After they had watched the videos, we asked the participants to give an overall satisfaction assessment with the virtual experience. Satisfaction was measured on a numerical rating scale from 0 (not satisfied at all) to 10 (very satisfied), as used in other studies in the health sector (Aldosari et al., 2017). The overall rating was 7.45 points.

The analysis showed only a moderate inverse correlation between valence (SAM) at the end of video 3 (H) and overall satisfaction (*r* = −0.408). That is, lower valence (more joy) when the video ended is associated with higher overall satisfaction. The means of the correlations between valences after Stages 1 (PC) and 2 (SC), and overall satisfaction, were higher than 0.05, therefore we rejected the null hypothesis that significant differences exist (Table 3).

**Table 3:** Correlations between valence (SAM) and overall satisfaction.

Correlation between	Pearson correlation	Sig. (bilateral)
1. Valence (V1-PC) and overall satisfaction	0.009	0.952
2. Valence (V2-SC) and overall satisfaction	−0.109	0.457
3. Valence (V3-H) and overall satisfaction	−0.408	**0.004**

*Bold values represent the significant difference*.

### Facial Expression Analysis Results

**Figure 6:**
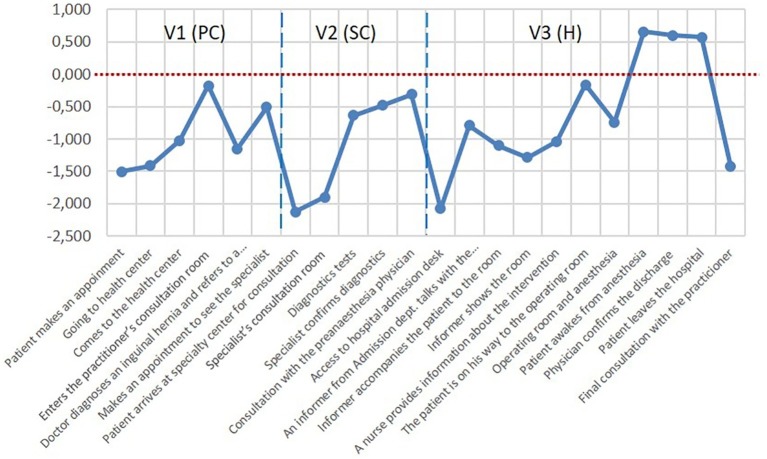
Valence indicator (FEA) through the patient journey.

The facial expression analysis (FEA) results confirmed the prevalence of negative valence in the emotional state of the participants during the *PJ*. In this respect, the results of the SAM questionnaires completed by the participants coincided with the measurements obtained through the FEA (Figure 6). The valence metric derived from the FEA showed negative values at the early stages of the care process. Of the 22 specific moments/points of the *PJ*, only three had positive valence: the scores became positive only after the surgical intervention was completed and up to discharge, which is consistent with the predominance of negative emotions in the healthcare experience.

At each stage, valence had more negative values but improved as the process moved forward, but access to a new care level (specialized care or hospitalization) returned emotional valence to low levels.

Once the need to go to the PC physician is recognized, the request for an appointment *via* the Internet has a negative value (−1.5). The first visual contact and the PC physician’s greeting improved valence to a neutral value (−0.175). The first diagnosis increased the negative value (−1.155), and the appointment request reduced it again (−0.511).

When attending the appointment with the specialist physician -after weeks of waiting- the entry to the center, the journey to the consultation room, and the wait to see the consultant, took valence to its most negative value (−2.123). The specialist care, the testing process and the return to the physician for confirmation of the diagnosis improved the valence value, but it always remained negative. The arrival at the operating theater and the process prior to the intervention also placed the valence in negative values.

At the recovery moment, the valence for the first time reached positive values (0.651); the values were also positive during the patient’s transfer to the recovery room (0.595) and at discharge from the hospital (0.568). During the subsequent PC check-up, the valence returned to the negative zone (−1.427), even lower than at the first contact with the general practitioner at the beginning of the *PJ* (−0.175).

Regarding the existence or lack thereof a correlation between the results of the valence reported by participants through the SAM questionnaire and those provided by the facial expression analysis (FEA), the results show no correlation at the end of each stage, as the significance values are above 0.05 (Table 4).

**Table 4:** Correlation between valence (SAM) and valence (FEA).

	V1 (PC)		V2 (SC)		V3 (H)	
Correlation between	Pearson correlation	Sig. (bi-lateral)	Pearson correlation	Sig. (bi-lateral)	Pearson correlation	Sig. (bi-lateral)
Valence (SAM) and valence (FEA)	−0.234	0.072	−0.049	0.709	−0.134	0.308

## Discussion and Conclusions

First, the results show the difference between the emotional valence of the patient’s experience reported through questionnaires and the valence obtained through neurophysiological data (analysis of facial expressions). Second, the results allow a contrast to be made of the validity of a new methodology to assess the affective dimension of the patient’s experience.

The present study confirms the existence of two dimensions identified in the literature on patient satisfaction: a cognitive type, demonstrated by data collected through a SAM questionnaire presented at the end of each stage of the process, and an affective type, obtained through the analysis of participants’ facial expressions during the experiment. The results showed that both series of data effectively address the valence concept but show differences between the dimensions. While questionnaires collect a cognitive, or a more rational evaluation of the experience, facial expression analysis provides an affective, or emotional, assessment.

In the quantification of satisfaction, it cannot be concluded which of the two components, cognitive or emotional, is the more important. However, it is certainly true that they are distinct dimensions and, thus, must both be considered, since they contribute differently to satisfaction.

It was observed that, although patients reported high levels of satisfaction in the questionnaires, the valence shown in the FEA was negative during the almost complete PJ, and reached positive values only as the time of discharge approached. The reasons for this may be diverse, from living an unwanted experience in an unknown environment to the influence of specific factors, such as communication with the professionals or the physical aspects of the facility.

The PJ analysis showed that the moments with the most negative valence values are those related to organizational aspects, during which there was no contact with medical staff, such as accessing the center, waits or requests for appointments, or admission procedures prior to hospitalization. When there is contact with health professionals, this index improves. This result highlights the role of other aspects beyond medical care, such as communication with the physician, which contribute decisively to the emotional state of the patient.

The correlation found between the valences reported by the SAM questionnaire at the end of the hospitalization phase and final satisfaction shows that the patients did not consider the negative aspects of the process, such as delays, in the assessment of overall satisfaction. This effect was also seen in Redelmeier and Kahneman (1996)’s study of patients undergoing colonoscopy: they concluded that people do not perceive the sum of an experience, but rather their average experience and how the process ended. When the experience ended positively, the patient had a better assessment than when it ended negatively.

With regard to the new methodology proposed, the PJ paradigm helped to reproduce the patient’s experience and collect, for the first time, neurophysiological data (facial expressions) during the care process, to achieve a broader view of the experience. This methodology can be applied in the study of other PJs, to improve specific processes and to analyze transversal issues, such as doctor-patient communication, and the influence of environmental aspects. It would be possible to apply it to other types of services.

In conclusion, it can be stated that, hitherto, the rational, or cognitive dimension has dominated in patient satisfaction assessments, and the affective or emotional dimension has been relegated to the background. For this reason, it seems appropriate now to incorporate this dimension to complete the satisfaction assessment. This will provide new ways of improving service and environmental processes and consequently increase customer satisfaction in health services.

Regarding future lines of study, it will be necessary to study in greater depth the emotional aspect of the patient’s experience to complement the techniques currently used with others that help to explain the affective dimension, such as FEA. Finally, it is acknowledged that the present study has limitations. First, the sample size is not large, although it exceeds some used in other studies employing these types of techniques. Second, it should be noted that the proposal for a new methodology, i.e., FEA, carried out in the laboratory to analyze emotional valence, has no background use in healthcare through which to compare the results. Third, the characteristics of the chosen PJ, which cover a particular experience, mean that the results should not be generalized.

## Data Availability Statement

The datasets generated for this study are available on request to the corresponding author.

## Ethics Statement

The studies involving human participants were reviewed and approved by Hospital of Alcalá de Henares, Madrid. The patients/participants provided their written informed consent to participate in this study.

## Author Contributions

All authors have participated in all stages of work, including the conception and design of the research, the revision of intellectual content, and drafting the work.

### Conflict of Interest

The authors declare that the research was conducted in the absence of any commercial or financial relationships that could be construed as a potential conflict of interest.

## References

[ref1] Aldosari M. A. Tavares M. A. Matta-Machado A. T. A. Abreu M. H. (2017). Factors associated with patients’ satisfaction in Brazilian dental primary health care. PLoS One 12:e0187993. 10.1371/journal.pone.0187993, PMID: 29145438 PMC5690593

[ref2] Ausin J. M. Guixeres J. Bigné E. Alcañiz M. (2017). “Facial expressions to evaluate advertising: a laboratory versus living room study” in Advances in advertising research, VIII. eds. Zabkar V. Eisend M. (Germany: Gabler Verlag), 109–122.

[ref3] Bendall-Lyon D. Powers T. L. (2004). The impact of structure and process attributes on satisfaction and behavioral intentions. J. Serv. Mark. 18, 114–121. 10.1108/08876040410528719

[ref4] Bradley M. M. Lang P. J. (1994). Measuring emotion: the self-assessment Manikin and the semantic differential. J. Behav. Ther. Exp. Psychiatry 25, 49–59. 10.1016/0005-7916(94)90063-9, PMID: 7962581

[ref5] Bruno A. Dell’Aversana G. Zunino A. (2017). Customer orientation and leadership in the health service sector: the role of workplace social support. Front. Psychol. 8:1920. 10.3389/fpsyg.2017.01920 29163297 PMC5672502

[ref6] Cacioppo J. Gardner W. (1999). Emotion. Annu. Rev. Psychol. 50, 191–214. 10.1146/annurev.psych.50.1.191, PMID: 10074678

[ref8] Chih-Chien W. Ming-Chang H. (2014). An exploratory study using inexpensive electroencephalography (EEG) to understand flow experience in computer-based instruction. Inf. Manag. 51, 912–923. 10.1016/j.im.2014.05.010

[ref9] Coulter A. Locock L. Ziebland S. Calabrese J. (2014). Collecting data on patient experience is not enough: they must be used to improve care. BMJ 348:g2225. 10.1136/bmj.g2225 24671966

[ref10] Cronin J. J. Taylor S. A. (1992). Measuring service quality. A reexamination and extension. J. Mark. 56, 55–68.

[ref11] Crow R. Gage H. Hampson S. Hart J. Kimber A. Storey L.. (2002). The measurement of satisfaction with health care: implications for practice from a systematic review of the literature. Health Technol. Assess. 6, 1–244. 10.3310/hta6320, PMID: 12925269

[ref12] Dubé L. Menon K. (1998). Managing emotions. Mark. Health Serv. 18, 35–42. 10185307

[ref13] Ekman P. Friesen W. V. (1971). Constants across cultures in the face and emotion. J. Pers. Soc. Psychol. 17, 17124–17129. 10.1037/h0030377 5542557

[ref14] Ekman P. Friesen W. V. (1978). Manual for the facial action coding system. Palo Alto, California: Consulting Psychologists Press.

[ref15] Frijda N. H. Mesquita B. (1998). “The analysis of emotions” in What develops in emotional development? eds. Mascolo M. F. Griffin S. (New York: Plenum Press), 273–295.

[ref16] Fulbright R. K. Troche C. J. Skudlarski P. Gore J. C. Wexler B. E. (2001). Functional MR imaging of regional brain activation associated with the affective experience of pain. AJR Am. J. Roentgenol. 177, 1205–1210. 10.2214/ajr.177.5.1771205 11641204

[ref17] Gentile C. Spiller N. Noci G. (2007). How to sustain the customer experience: an overview of experience components that co-create value with the customer. Eur. Manag. J. 25, 395–410. 10.1016/j.emj.2007.08.005

[ref18] Giese J. L. Cote J. A. (2000). Defining consumer satisfaction. Acad. Mark. Sci. Rev. 1, 1–34.

[ref19] Gill L. White L. (2009). A critical review of patient satisfaction. Leadersh. Health Serv. 22, 8–19. 10.1108/17511870910927994

[ref20] Granado S. Rodríguez C. Olmedo M. Chacón A. Vigil D. Rodríguez P. (2007). Diseño y validación de un cuestionario para evaluar la satisfacción de los pacientes atendidos en las consultas externas de un hospital de Madrid en 2006. Rev. Esp. Salud Publica 81, 637–645. 10.1590/S1135-57272007000600007 18347747

[ref21] Greenwald M. K. Cook E. W. Lang P. J. (1989). Affective judgment and psychophysiological response: dimensional covariation in the evaluation of pictorial stimuli. J. Psychophysiol. 3, 51–64.

[ref22] Handayani D. Wahab A. Yaacob H. (2015). Recognition of emotions in video clips: the self-assessment Manikin validation. Telkomnika 13, 1343–1351. 10.12928/telkomnika.v13i4.2735

[ref23] Hunt H. K. (1977). “CS/D overview and future research directions” in Conceptualization and measurement of consumer satisfaction and dissatisfaction. ed. Hunt K. (Cambridge, MA: Marketing Science Institute), 455–488.

[ref24] iMotions (2018). iMotions. Biometric Research. Available at: https://imotions.com/facial-expressions/ (Accessed July 20, 2018).

[ref26] Jayanti R. K. Whipple T. W. (2008). Like me... like me not: the role of physician likability on service evaluations. J. Mark. Theory Pract. 16, 79–86. 10.2753/MTP1069-6679160106

[ref27] Lees C. (2011). Measuring the patient experience. Nurs. Res. 19, 25–28. 10.7748/nr2011.10.19.1.25.c8768, PMID: 22128584

[ref28] Liljander V. Strandvik T. (1997). Emotions in service satisfaction. Int. J. Serv. Ind. Manag. 8, 148–169. 10.1108/09564239710166272

[ref29] Losada M. Rodríguez A. (2007). Calidad del servicio de salud: una revisión a la literatura desde la perspectiva del marketing. Cuad. Adm. 20, 237–258.

[ref30] Mehrabian A. (1996). Pleasure-arousal-dominance: a general framework for describing and measuring individual differences in temperament. Curr. Psychol. 14, 261–292. 10.1007/BF02686918

[ref31] Mehrabian A. Russell J. A. (1974). An approach to environmental psychology. Cambridge: MIT Press.

[ref33] Ofili O. U. (2014). Patient satisfaction in healthcare delivery. A review of current approaches and methods. Eur. Sci. J. 10, 25–39.

[ref34] Oliver R. L. (1993). Cognitive, affective, and attribute bases of the satisfaction response. J. Consum. Res. 20, 418–430. 10.1086/209358

[ref36] Pelegrín-Borondo J. Juaneda-Ayensa E. González-Menorca L. González-Menorca C. (2015). Dimensions and basic emotions. A complementary approach to the emotions produced to tourists by the hotel. J. Vacat. Mark. 21, 351–365. 10.1177/1356766715580869

[ref37] Phillips D. Baumgartner H. (2002). The role of consumption emotions in the satisfaction response. J. Consum. Psychol. 12, 243–252. 10.1207/S15327663JCP1203_06

[ref38] Posner M. I. Rothbart M. K. (1998). “Summary and commentary: developing attentional skills” in Cognitive neuro-science of attention: A developmental perspective. ed. Richards J. E. (Mahwah, NJ: Erlbaum), 317–323.

[ref39] Redelmeier D. A. Kahneman D. (1996). Patients’ memories of painful medical treatments: real-time and retrospective evaluations of two minimally invasive procedures. Pain 66, 3–8. 10.1016/0304-3959(96)02994-6, PMID: 8857625

[ref40] Reeve J. (2015). Understanding motivation and emotion. New York: Wiley.

[ref41] Rundle-Thiele S. Russell-Bennett R. (2010). Patient influences on satisfaction and loyalty for GP services. Health Mark. Q. 27, 195–214. 10.1080/07359681003745162 20446141

[ref42] Russell J. A. (1980). A circumplex model of affect. J. Pers. Soc. Psychol. 39, 1161–1178. 10.1037/h0077714

[ref43] Scherer K. (2005). What are emotions? And how can they be measured? Soc. Sci. Inf. 44, 695–729. 10.1177/0539018405058216

[ref44] Setz C. Schumm J. Lorenz C. Arnrich B. Tröster G. (2009). “Using ensemble classifier systems for handling missing data in emotion recognition from physiology: one step towards a practical system” in Affective computing and intelligent interaction and workshops, 2009. ACII 2009. 3rd International Conference (Amsterdam). 1–8, 10–12.

[ref45] Sofaer S. Firminger K. (2005). Patient perceptions of the quality of health services. Annu. Rev. Public Health 26, 513–559. 10.1146/annurev.publhealth.25.050503.153958, PMID: 15760300

[ref46] Suomala J. Palokangas L. Leminen S. Westerlund M. Heinonen J. Numminen J. (2012). Neuromarketing: understanding customers’ subconscious responses to marketing. Technol. Innov. Manag. Rev. 2, 12–21. 10.22215/timreview/634

[ref47] The Beryl Institute (2015). State of patient experience 2015: A global perspective on the patient experience. Available at: http://www.theberylinstitute.org/?page=Mission (Accessed June 12, 2015).

[ref48] The King’s Fund (2010). A high performing NHS? A review of Progress 1997–2010. London: The King’s Fund.

[ref49] Val-Jiménez C. L. López-Torres J. García-Atienza E. M. Navarro-Ruiz M. S. Hernández-Cerón I. Moreno de la Rosa L. (2017). Satisfacción con el tratamiento en pacientes de Atención Primaria con artrosis. Rev. Esp. Salud Publica 91, e1–e10. PMC11587334 28682304

[ref50] Vance A. Eargle D. Anderson B. B. Kirwan C. B. (2014). Using measures of risk perception to predict information security behavior: insights from electroencephalography (EEG). J. Assoc. Inf. Syst. 15, 679–722. 10.17705/1jais.00375

[ref51] Villodre R. Calero R. González-Gallarza M. (2014). La satisfacción del cliente como indicador de calidad en neurorehabilitación. Cuad. Estud. Empres. 24, 131–147. 10.5209/rev_CESE.2014.v24.48614

[ref52] Vinagre M. H. Neves J. (2008). The influence of service quality and patients’ emotions on satisfaction. Int. J. Health Care Qual. Assur. 21, 87–103. 10.1108/09526860810841183, PMID: 18437942

[ref53] Westbrook R. A. Oliver R. L. (1991). The dimensionality of consumption emotion patterns and consumer satisfaction. J. Consum. Res. 18, 84–91. 10.1086/209243

[ref54] Wirtz J. Bateson J. (1999). Consumer satisfaction with services: integrating the environment perspective in services marketing into the traditional disconfirmation paradigm. J. Bus. Res. 44, 55–66. 10.1016/S0148-2963(97)00178-1

[ref55] Wolf J. A. Niederhauser V. Mashburn D. LaVela S. L. (2014). Defining patient experience. Patient Exp. J., 17–19. 10.35680/2372-0247.1004

